# Impact of Time-Restricted Feeding to Late Night on Adaptation to a 6 h Phase Advance of the Light-Dark Cycle in Mice

**DOI:** 10.3389/fphys.2021.634187

**Published:** 2021-02-16

**Authors:** Baoyin Ren, Changxiao Ma, Lihong Chen, Garret A. FitzGerald, Guangrui Yang

**Affiliations:** ^1^School of Bioengineering, Dalian University of Technology, Dalian, China; ^2^Advanced Institute for Medical Sciences, Dalian Medical University, Dalian, China; ^3^Perelman School of Medicine, Institute for Translational Medicine and Therapeutics, University of Pennsylvania, Philadelphia, PA, United States

**Keywords:** time-restricted feeding, behavior, adaptation, sepsis, jet lag

## Abstract

In modern society, more and more people suffer from circadian disruption, which in turn affects health. But until now, there are no widely accepted therapies for circadian disorders. Rhythmic feeding behavior is one of the most potent non-photic zeitgebers, thus it has been suggested that it was important to eat during specific periods of time (time-restricted feeding, TRF) so that feeding is aligned with environmental cues under normal light/dark conditions. Here, we challenged mice with a 6 h advanced shift, combined with various approaches to TRF, and found that food restricted to the second half of the nights after the shift facilitated adaptation. This coincided with improved resilience to sepsis. These results raise the possibility of reducing the adverse responses to jet lag by subsequent timing of food intake.

## Introduction

Despite an increasing awareness of the hazards of circadian disruption, modern lifestyle is subject to frequent desynchronization due to shift working, artificial light, and transmeridian air flight. While the molecular clock may be adjusted by small molecules that target clock proteins (Cho et al., 2012; Solt et al., 2012; Wallach and Kramer, 2015), we have found that inhibiting circadian rhythms may also mitigate circadian disorders (Yang et al., 2019, 2020).

As a potent non-photic cue, food is an important secondary zeitgebers in animal models (Golombek and Rosenstein, 2010). Unlike light, daily feeding behavior mainly affects peripheral clocks, especially in the liver (Damiola et al., 2000; Hara et al., 2001; Stokkan et al., 2001; Fuller et al., 2008). Indeed, studies in rodents suggest the benefits of eating during specific periods of time (time-restricted feeding, TRF) so that feeding is aligned with light cues. For example, if food is available only at night, during the active phase in mice, it can prevent the metabolic syndrome induced by a high-fat diet (Hatori et al., 2012), and even reverse pre-existing obesity and impaired glucose tolerance (Chaix et al., 2014). These beneficial effects are lost on reversion to more temporally disrupted *ad libitum* feeding.

Although there is evidence of a beneficial effect of TRF on health under regular light/dark conditions, it is not known if TRF could still retain its advantage under disrupted time schedules, such as jet lag. When animals are challenged with artificial jet lag, the central clock can be quickly reset by the new light/dark cycle, while the peripheral clocks adjust over a longer time scale (Yamazaki et al., 2000; Kiessling et al., 2010). This temporal lag represents the key determinant of adjustment to jet lag. Since feeding behavior can affect peripheral clocks, certain TRF regimens would facilitate adaptation to jet lag. Accordingly, feeding scheduled to the first 2 h in dark phase accelerated re-entrainment speed in rats treated with a 6 h advanced jet lag (Angeles-Castellanos et al., 2011; Ubaldo-Reyes et al., 2017). Similarly, a recent report showed that restricting food intake to the whole dark phase (12 h) accelerated adjustment to weekly repeated day-night reversals in female FVB (Friend Leukemia Virus B) mice (Schilperoort et al., 2019). Despite the fact that activity data reflect the speed of adjustment to a jet lag, it does not necessarily mean faster responders are healthier. Objective evaluation of health status has to be taken into account when describing circadian adaptation. Although chronic circadian disruption (such as multiple jet lags) has been related to various diseases in human and animal models (Yang et al., 2013), a single jet lag is too mild to cause obvious health problems in normal mice. Interestingly, a bacterial lipopolysaccharide (LPS)-induced sepsis in mice was exacerbated after four 6 h advanced shifts or showed a statistically non-significant trend toward to exacerbation after a single shift (Castanon-Cervantes et al., 2010). This effect was evident even 7 days after the last shift when mice were fully re-entrained. Therefore, in our study, we utilized this jet lag sensitive disease model to investigate if any TRF-facilitated adaptation to a 6 h advanced shift was accompanied by resilience to LPS.

To simplify the study, we chose the combination of various 6 h TRFs and a 6 h advanced shift (Supplementary Figure S1), the most commonly used artificial jet lag, as the first attempt to explore the possible beneficial effect of TRF under circadian disruption conditions. The 6 h TRF was set to the first or the second half of the nights, so they can cover the whole night without leaving a gap. Immediately after the behavioral study, mice were treated with LPS to induce sepsis for evaluation of health status. We used these approaches to determine whether any strategies might facilitate adaptation to the jet lag.

## Materials and Methods

### Mice

Eightweek-old male C57BL/6 mice were individually housed in well-ventilated and light-proof cabinet (Probecare, Wuhan, China). Mice were kept under regular light/dark cycles (12 h light:12 h dark, LD) at room temperature for 2 weeks before experiments, during which all mice had free access to normal chow diet (Changsheng Biotechnology, Benxi, China) and water. Lights-on was at 08:00 h, defined as zeitgeber time 0 (ZT0), and lights off at 20:00 h defined as ZT12. Light intensity at the level of animals during the day was about 100 lux.

### Study Design

Two sets of mice were treated with or without a 6 h advanced shift, combined with a variety of TRF regimens. The mice in the first set (Supplementary Figure S1) were given food in the first (ZT12-18) or the second half (ZT18-0) of the night 1 week before and after the shift (group B, C, D, E) or have free access to food (group A). Another set of mice were divided into three groups (Supplementary Figure S3), i.e., the control group (no jet lag, no TRF); the group of jet lag without TRF; and group of jet lag with TRF (TRF was set to the second half of the nights after the shift).

We used this approach to investigate the speed of adaptation to the 6 h advanced shift. 7 days after the shift, mice were peritoneally injected with LPS (Solarbio, Beijing, China) to induce sepsis (Castanon-Cervantes et al., 2010). The health status and lethality were then monitored for a week, during which food and water bottle were placed on bedding for convenient access without TRF.

### Wheel Running Activity

Mice for activity recording were individually housed in a running wheel-equipped cage contained within the cabinet described above. The wheel revolution was continuously recorded throughout the experiment and analyzed using ClockLab software (Actimetrics, Wilimette, IL, United States). The number of days until stable re-entrainment of the activity rhythm following the shift was determined as previously described (Pfeffer et al., 2015) with modification. Specifically, entrainment to the new LD cycle was defined as activity onset relative to lights off <0.5 h for 4 consecutive days or for days before LPS treatment if less than 4. A mouse not re-entrained 7 days after the shift was considered as re-entrained on day 8 for statistical analysis.

### Sepsis Model

Mice were injected intraperitoneally with LPS (1.5 mg/ml in PBS, 10 μL/g body weight, i.e., 15 mg/kg body weight) at ZT3. Health status was observed every 3 h for the first 2 days, and then every 6 h for 5 more consecutive days. The health status was scored as previously described with modifications (Shrum et al., 2014), including the level of consciousness, the response to stimulation, and the survival rate, as follows (Table 1).

**TABLE 1:** Sepsis score.

Score	Level of consciousness	Response to stimulus
1	Mouse is active but avoids standing upright	Slow or no response to auditory stimulus; strong response to touch (moves to escape)
2	Mouse activity is noticeably slowed. The mouse is still ambulant	No response to auditory stimulus; moderate response to touch (moves a few steps)
3	Activity is impaired. Mouse only moves when provoked, movements have a tremor	No response to auditory stimulus; mild response to touch (no locomotion)
4	Activity severely impaired. Mouse remains stationary when provoked, with possible tremor	No response to auditory stimulus. Little or no response to touch. Cannot right itself if pushed over
5	Death	Death

### Statistics

All statistical tests were two-sided. Student’s *t-*test was used when a single variable was compared between two groups. One-way or 2-way ANOVA with Tukey’s test was used for multiple comparisons. The log-rank test was used to compare the survival distributions. In all figures with error bars, the graphs depict means ± SEM.

## Results

### Effect of TRF on the Behavior of Jet Lagged Mice

**FIGURE 1:**
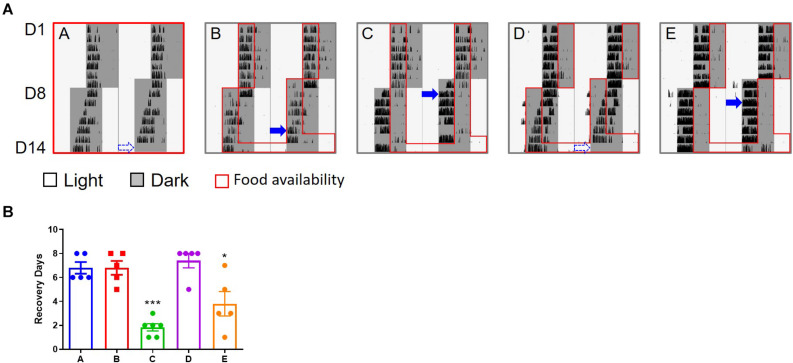
Wheel running activity of mice under 6 h advanced jet lag. **(A)** Representative double–plotted actograms of wheel running activity of mice in group A–E. Blue arrows indicate the date by which the mice were re-entrained to the new LD cycle. The dashed arrows represent that the mice did not adapt to the new schedule within 7 days and were considered re-entrained on day 8. **(B)** Days for recovery from jet lag. ***p < 0.001, C vs. A, B, or D; *p < 0.05, E vs. A, B, or D. There is no statistical difference between C and E, or between any two groups among A, B, and D. One-way ANOVA with Tukey’s multiple comparisons test.

Mice (groups C and E) whose food was only available late at night after the 6 h advanced shift adjusted much faster than those in the other three groups (Figure 1 and Supplementary Figure S2). These were the mice not subjected to TRF (group A, the control group), and the mice with TRF during the first half of the nights after the shift (groups B and D). On average, the mice in groups C and E recovered from the shift in 2–3 days based on the calculation of their onset of daily activity, while about 7 days was required for the slow responders (group A, B, and D) to adapt to the change of the schedule.

### Resilience in Septic Mice

**FIGURE 2:**
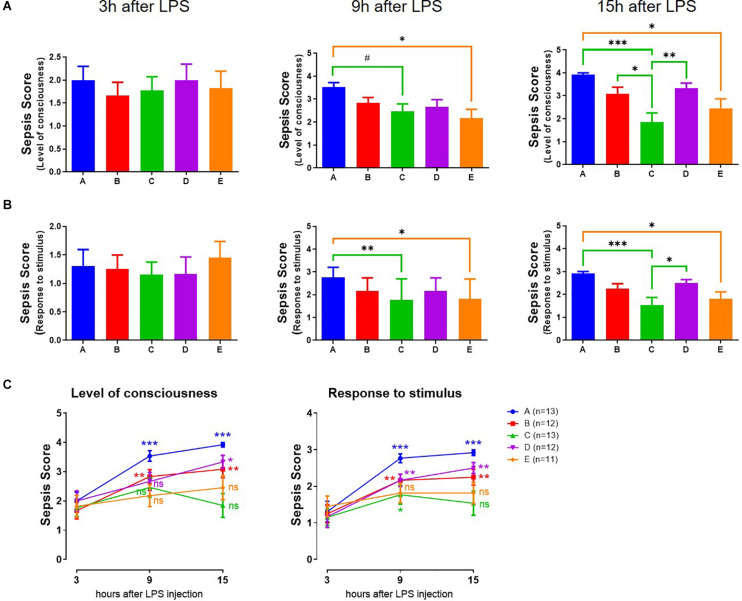
Assessment of severity of LPS-induced sepsis (sepsis score). **(A)** Level of consciousness (*n* = 11–13). **(B)** Response to auditory stimulus and touch. ***p < 0.001; **p < 0.01; *p < 0.05; #*p* < 0.1, One-way ANOVA with Tukey’s multiple comparisons test (*n* = 11–13). **(C)** Comparison between time points. ***p < 0.001; **p < 0.01; *p < 0.05; ns, no statistical difference, 9 or 15 h vs. 3 h, paired Student’s *t*-test.

To test if the speed of adaptation is correlated with their health status, we injected the mice with LPS to induce sepsis and test their resilience to a perturbation. The end points were the level of consciousness (Figure 2A) or the response to an auditory or tactile stimulus (Figure 2B). Together these aggregated to a sepsis score. This was evident 3 h after LPS, without differences in the groups (Figure 2). However, at 9 and 15 h after LPS injection, the sepsis score in mice in groups A, B, and D (the slow responders to the jet lag) had further deteriorated (Figure 2C), while that in the fast responders (group C and E) remained constant level.

### Survival of Septic Mice

**FIGURE 3:**
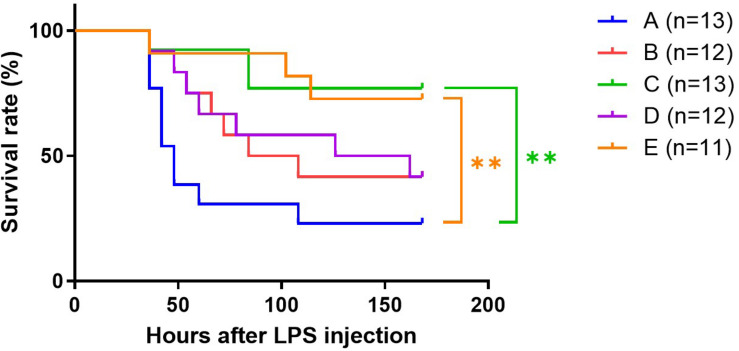
Effect of TRF on survival of septic mice. **p < 0.01, Log-rank test.

The control group (Group A) had more fatal events than group C and E, the fast responders (Figure 3). One week after LPS treatment only 23% survived in Group A whereas more than 70% survived in groups C and E (*p* < 0.01). The survival rate of the mice in groups B and D was 42%; both had a trend toward a decrease in survival rate compared with group C (B vs. C, *p* = 0.066; D vs. C, *p* = 0.074).

### The Behavior of Mice After LPS Treatment

**FIGURE 4:**
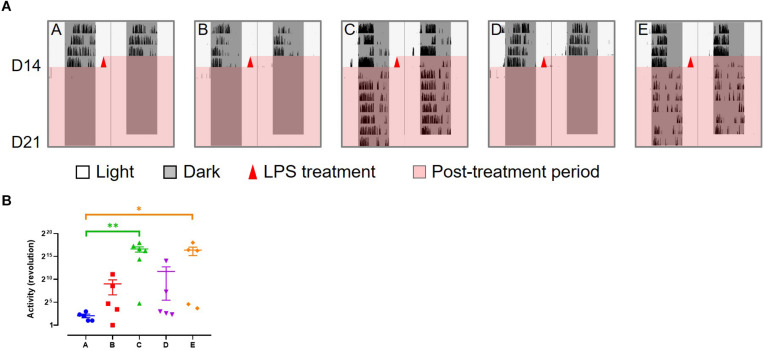
Wheel running activity of LPS-treated mice. **(A)** Representative wheel running activity after LPS treatment. **(B)** Total number of wheel revolution after LPS treatment. Data are presented as mean ± SEM. Dunn’s multiple comparisons test, *p < 0.05; **p < 0.01.

Wheel running activity was continuously recorded throughout the experiment. After LPS treatment, there was little activity in the slow responders (group A, B, and D), while the fast responders (group C and E) recovered to a certain level after a short period of mild disturbance (Figure 4). These changes are consistent with their health status.

### Additional Study With TRF After Phase Shift Only

These results suggested that making food available late at night after a 6 h advanced shift was beneficial, irrespective of setting the TRF early or late in the night phase before the shift. Thus, we performed additional experiment with TRF applied after the shift only.

**FIGURE 5:**
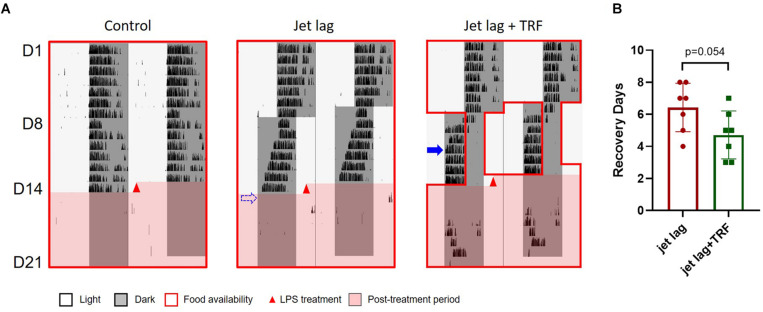
Wheel running activity of mice (the 2nd set). **(A)** Representative wheel running activity. Blue arrow indicates the date by which the mouse was re-entrained to the new LD cycle. The dashed arrow represents that the mouse did not adapt to the new schedule within 7 days and was considered re-entrained on day 8. **(B)** Total number of wheel revolution after LPS treatment. Data are presented as mean ± SEM. Dunn’s multiple comparisons test.

**FIGURE 6:**
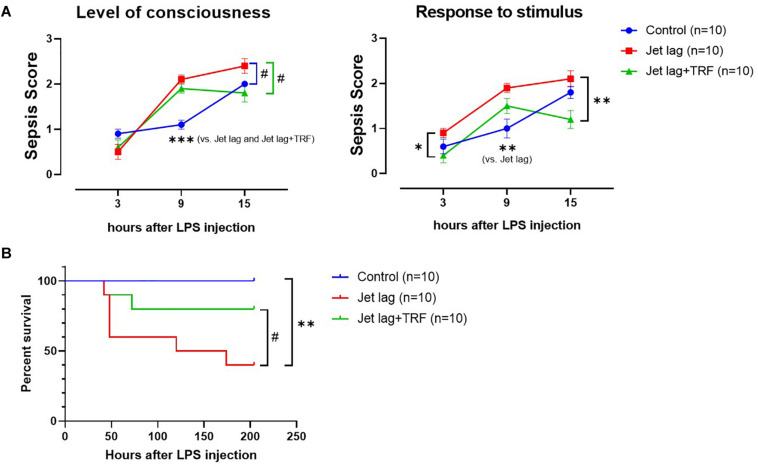
Effect of TRF on severity and survival of septic mice (the 2nd set). **(A)**, Assessment of severity of LPS-treated mice (sepsis score). Left, level of consciousness; right, response to auditory stimulus and touch. ***p < 0.001; **p < 0.01; ^#^*p* < 0.1, 2-way ANOVA with Tukey’s multiple comparisons test. **(B)** Effect of TRF on survival of septic mice. **p < 0.01; ^#^*p* < 0.1, Log-rank test.

Mice administered TRF late at night after the advanced shift adjusted their behavior faster than the mice without TRF (4vs. 6 days averagely, Figure 5 and Supplementary Figure S4). Similarly, subsequent TRF ameliorated sepsis score (Figure 6A), and survival rate (Figure 6B) after LPS treatment in jet lagged mice.

## Discussion

Disruption of circadian rhythms is associated with adverse health effects in humans and is causative of physiological dysfunction in rodent models (Yang et al., 2013). Despite the commonality of this phenomenon in modern life—up to a third of the workforce may pursue shift work—there are no strategies to alleviate such circadian disorders. Here, we speculated that timing of feeding behavior, one of the most potent non-photic zeitgebers, might offer such a possibility.

The benefits of TRF in rodent models have attracted much attention (Salgado-Delgado et al., 2010; Oike et al., 2015; Casiraghi et al., 2016; Longo and Panda, 2016; Chaix et al., 2019; Manoogian et al., 2019; Kriebs, 2020), although its value as a strategy in humans remains to be established. However, most studies were performed under regular light/dark conditions rather than disrupted light schedules. Recently, Schilperoort et al. (2019), exposed female FVB mice to weekly 12 h shifts (day-night reversal), combined with TRF for 28 weeks, and found that restricting food intake to the dark phase enhanced adaptation to the repeated shifts, as reflected by accelerated adjustment of core body temperature and activity rhythms. However, it seemed that TRF strategy did not improve the plasma lipids compared to *ad libitum* feeding. Perhaps the disruption induced by the jet lag paradigm or the benefit of food restricted to the whole night was insufficient to detect a difference. Additionally, the mice in this study were subjected to complete food restriction for 24 h each of the 28 weeks, an unusual feature in TRF protocols. There are also reports using rats showing that feeding restricted to the first 2 h in dark phase post a 6 h advanced shift accelerated re-entrainment speed in terms of behavior (Angeles-Castellanos et al., 2011; Ubaldo-Reyes et al., 2017; Escobar et al., 2020). However, none of them investigated if their TRF strategies have beneficial effect on health that may be compromised by a single jet lag.

In the current study, we investigated both behavioral adaptation and health status. Firstly, we used male mice to study the effect of TRF (during the dark phases only) on adaptation to a single jet lag (a 6 h advanced shift of light/dark cycle). We did not perform food restriction to the light phase either before or after the shift, as that could, respectively, introduce forced desynchrony (Damiola et al., 2000) or retard adaptation to jet lag. Usually, the rate of resynchronization of clock time and circadian time is 1–1.5 h/d (Weingarten and Collop, 2013). Initially, we presumed that the mice that had food available during the first half of the nights after a 6 h advanced shift would adapt to the time change quickly, because the availability of food would keep them awake. However, these mice took about 7 days to adjust their behavior, which is not distinguishable from jet-lagged mice without TRF. In contrast, when food was restricted to the second half of the nights after the shift, the mice adjusted more quickly—in only 2–3 days. One possible reason is that food-anticipatory activity is induced at the first half of the dark phase when food is available in the late night (Abe et al., 1989; Mistlberger, 2009).

However, faster adaptation to jet lag is not synonymous with avoidance of the adverse effects of circadian disruption. Many jet-lagged people have experience of forced adjustment which seems to be fast but may be accompanied by more symptoms of circadian disorders. Therefore, health status should be evaluated objectively when performing behavioral study. Thus, we utilized the jet lag sensitive LPS sepsis model and found that the speed of behavioral adaptation was highly correlated with resilience to LPS administration as reflected by septic score and lethality. Since the second half of the nights after the 6 h advanced shift can be regarded as subjective night following pre-shift periods, mice under such schedule will not be forced by food availability. This also suggests that the beneficial effect of TRF on adaptation to circadian disruption is time dependent.

In summary, a TRF strategy is described that accelerates adaptation to jet lag and restrains a measure of its adverse consequences in mice. Although we did not perform thoroughly studies by extending strategies of jet lag or TRF, our results raised a feasibility of timing of food intake to alleviate circadian clock disorders. This offers an approach to trans-meridian travel that might be tested, its extension to chronic models of circadian disruption might offer an approach to reducing the burden of cardiometabolic disease that has been associated with shift work in humans (Chen and Yang, 2015).

## Data Availability Statement

The original contributions presented in the study are included in the article/Supplementary Material, further inquiries can be directed to the corresponding author/s.

## Ethics Statement

The animal study was reviewed and approved by the Dalian University of Technology Institutional Animal Care and Use Committee.

## Author Contributions

GY and LC conceived and designed the research. BR performed all experiments. BR, CM, and GY analyzed the data. BR, LC, GF, and GY wrote the manuscript. All authors contributed to the article and approved the submitted version.

## Conflict of Interest

GF was a senior advisor to Calico Laboratories. The remaining authors declare that the research was conducted in the absence of any commercial or financial relationships that could be construed as a potential conflict of interest.
