# Supplementary material for: Impact of Time-Restricted Feeding to Late Night on Adaptation to a 6 h Phase Advance of the Light-Dark Cycle in Mice
Source: Front Physiol. 2021 Feb 16;12:634187. doi: 10.3389/fphys.2021.634187 (PMC7920952; doi:10.3389/fphys.2021.634187)
Supplement: Supplementary file 1 [file Presentation_1.PPTX]

## Slide 1
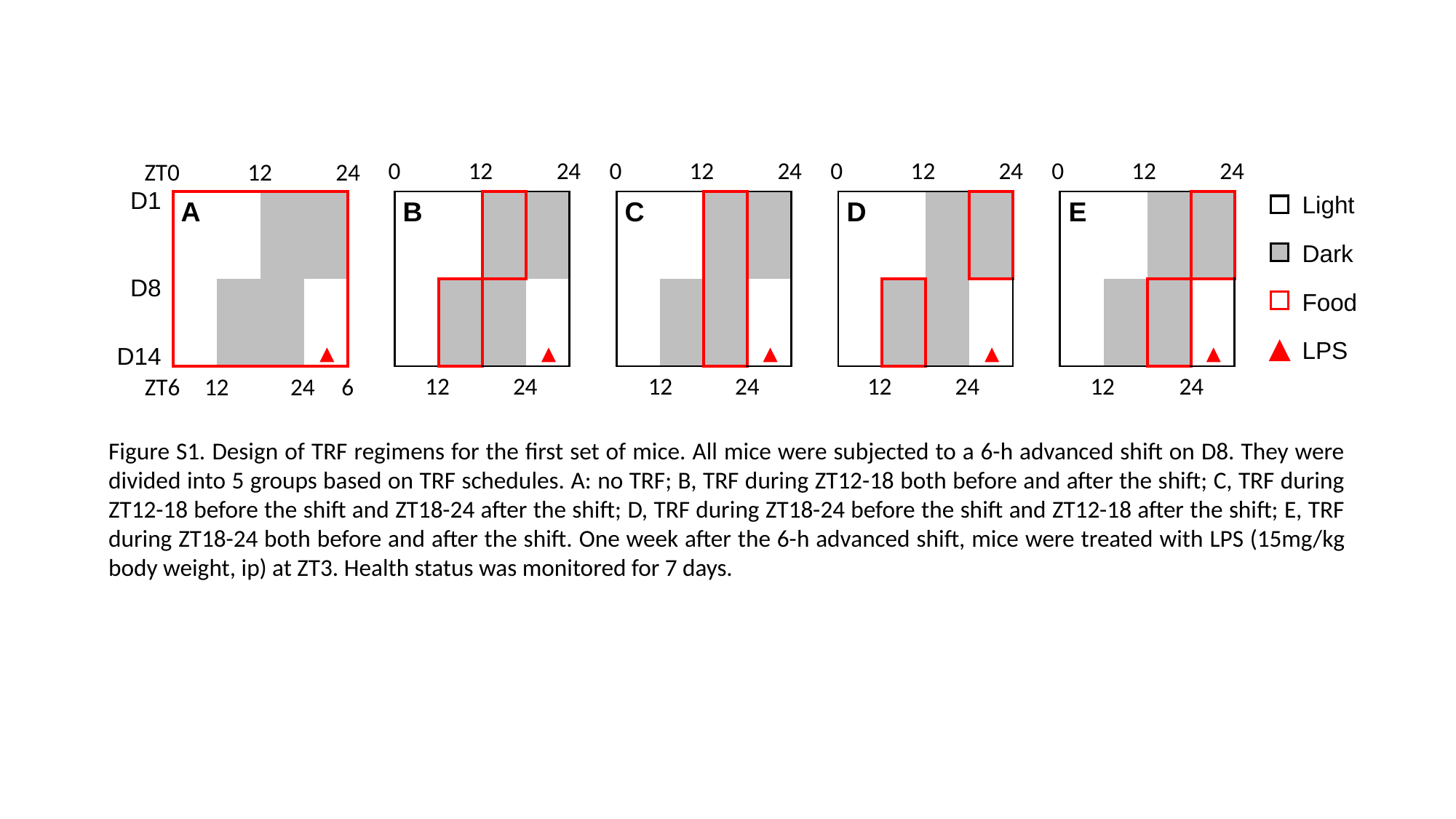

0
12
24
12
24
0
12
24
12
24
0
12
24
12
24
0
12
24
12
24
ZT0
12
24
ZT6
12
24
6
D1
Light
A
B
C
D
E
| | | | |
| --- | --- | --- | --- |
| | | | |
| | | | |
| | | | |
| | | | |
| --- | --- | --- | --- |
| | | | |
| | | | |
| | | | |
| | | | |
| --- | --- | --- | --- |
| | | | |
| | | | |
| | | | |
| | | | |
| --- | --- | --- | --- |
| | | | |
| | | | |
| | | | |
| | | | |
| --- | --- | --- | --- |
| | | | |
| | | | |
| | | | |
Dark
D8
Food
LPS
D14
Figure S1. Design of TRF regimens for the first set of mice. All mice were subjected to a 6-h advanced shift on D8. They were divided into 5 groups based on TRF schedules. A: no TRF; B, TRF during ZT12-18 both before and after the shift; C, TRF during ZT12-18 before the shift and ZT18-24 after the shift; D, TRF during ZT18-24 before the shift and ZT12-18 after the shift; E, TRF during ZT18-24 both before and after the shift. One week after the 6-h advanced shift, mice were treated with LPS (15mg/kg body weight, ip) at ZT3. Health status was monitored for 7 days.

## Slide 2
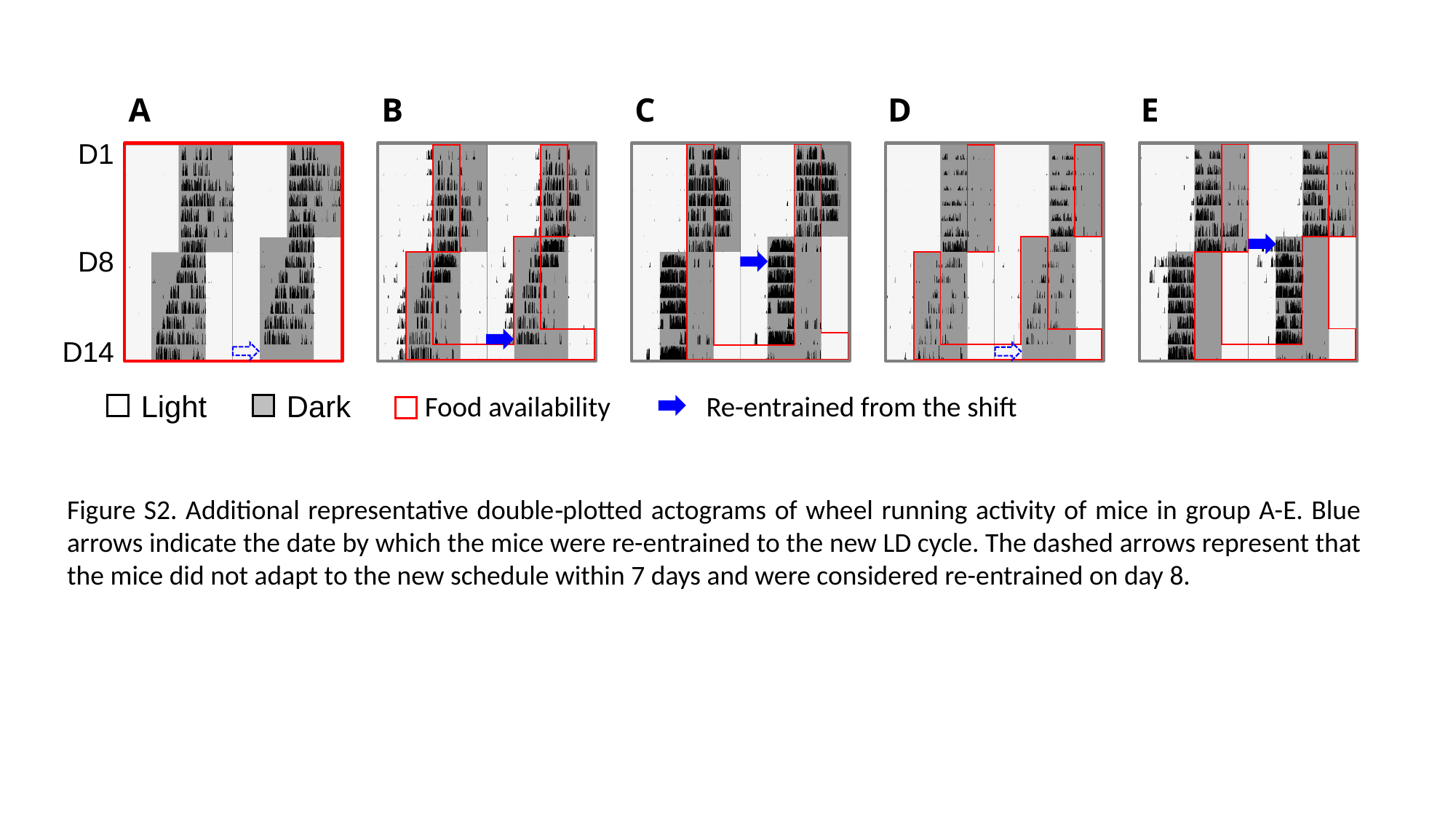

A
B
C
D
E
D1
| | | | | | | | |
| --- | --- | --- | --- | --- | --- | --- | --- |
| | | | | | | | |
| | | | | | | | |
| | | | | | | | |
| | | | | | | | |
| | | | | | | | |
| | | | | | | | |
| | | | | | | | |
| | | | | | | | |
| | | | | | | | |
| | | | | | | | |
| | | | | | | | |
| | | | | | | | |
| | | | | | | | |
| --- | --- | --- | --- | --- | --- | --- | --- |
| | | | | | | | |
| | | | | | | | |
| | | | | | | | |
| | | | | | | | |
| | | | | | | | |
| | | | | | | | |
| | | | | | | | |
| | | | | | | | |
| | | | | | | | |
| | | | | | | | |
| | | | | | | | |
| | | | | | | | |
| | | | | | | | |
| | | | | | | | |
| --- | --- | --- | --- | --- | --- | --- | --- |
| | | | | | | | |
| | | | | | | | |
| | | | | | | | |
| | | | | | | | |
| | | | | | | | |
| | | | | | | | |
| | | | | | | | |
| | | | | | | | |
| | | | | | | | |
| | | | | | | | |
| | | | | | | | |
| | | | | | | | |
| | | | | | | | |
| | | | | | | | |
| --- | --- | --- | --- | --- | --- | --- | --- |
| | | | | | | | |
| | | | | | | | |
| | | | | | | | |
| | | | | | | | |
| | | | | | | | |
| | | | | | | | |
| | | | | | | | |
| | | | | | | | |
| | | | | | | | |
| | | | | | | | |
| | | | | | | | |
| | | | | | | | |
| | | | | | | | |
D8
D14
Light
Dark
Food availability
Re-entrained from the shift
Figure S2. Additional representative double‐plotted actograms of wheel running activity of mice in group A-E. Blue arrows indicate the date by which the mice were re-entrained to the new LD cycle. The dashed arrows represent that the mice did not adapt to the new schedule within 7 days and were considered re-entrained on day 8.

## Slide 3
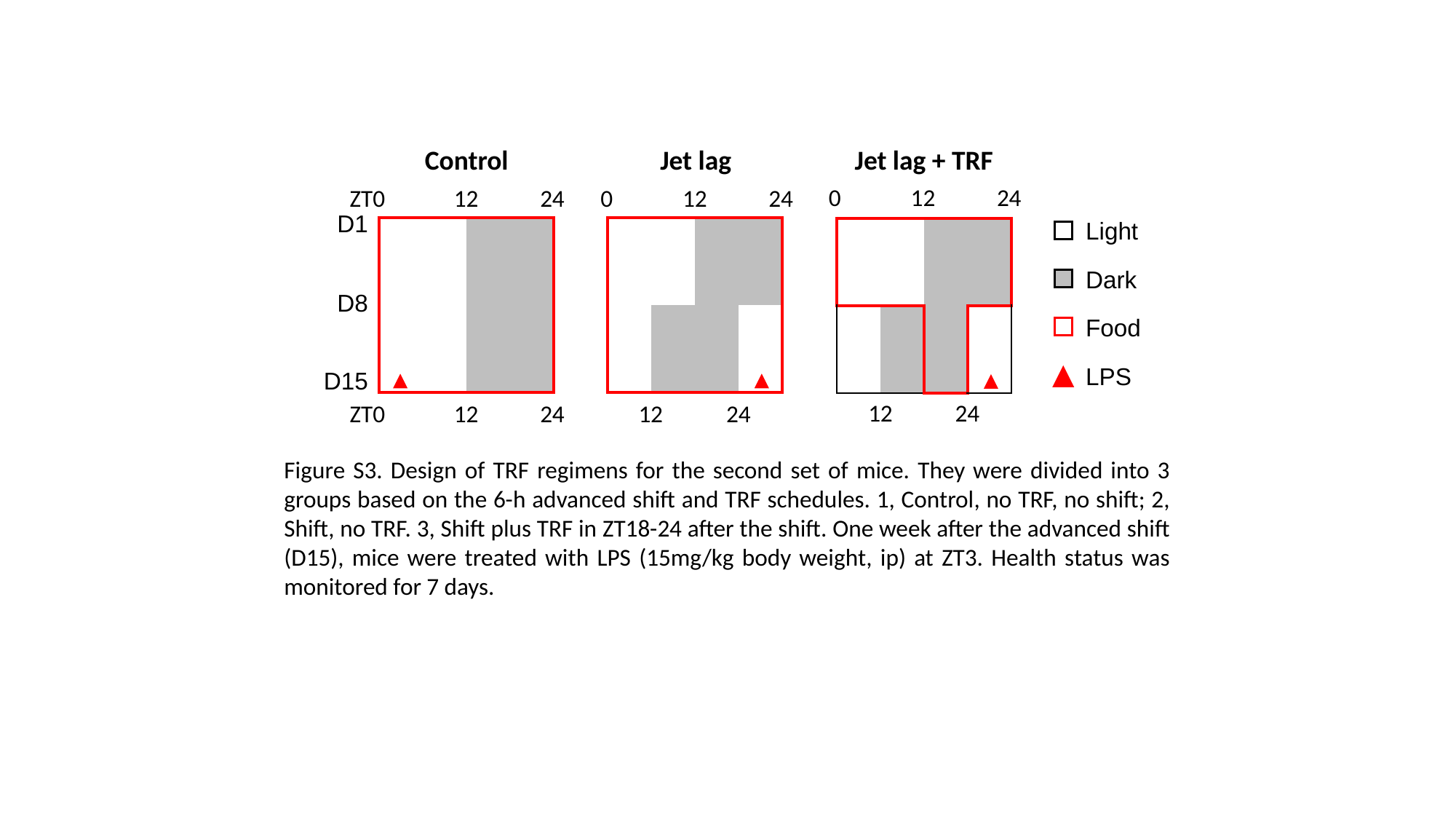

Control
Jet lag
Jet lag + TRF
0
12
24
12
24
ZT0
12
24
0
12
24
12
24
D1
Light
| | | | |
| --- | --- | --- | --- |
| | | | |
| | | | |
| | | | |
| | | | |
| --- | --- | --- | --- |
| | | | |
| | | | |
| | | | |
| | | | |
| --- | --- | --- | --- |
| | | | |
| | | | |
| | | | |
Dark
D8
Food
LPS
D15
ZT0
12
24
Figure S3. Design of TRF regimens for the second set of mice. They were divided into 3 groups based on the 6-h advanced shift and TRF schedules. 1, Control, no TRF, no shift; 2, Shift, no TRF. 3, Shift plus TRF in ZT18-24 after the shift. One week after the advanced shift (D15), mice were treated with LPS (15mg/kg body weight, ip) at ZT3. Health status was monitored for 7 days.

## Slide 4
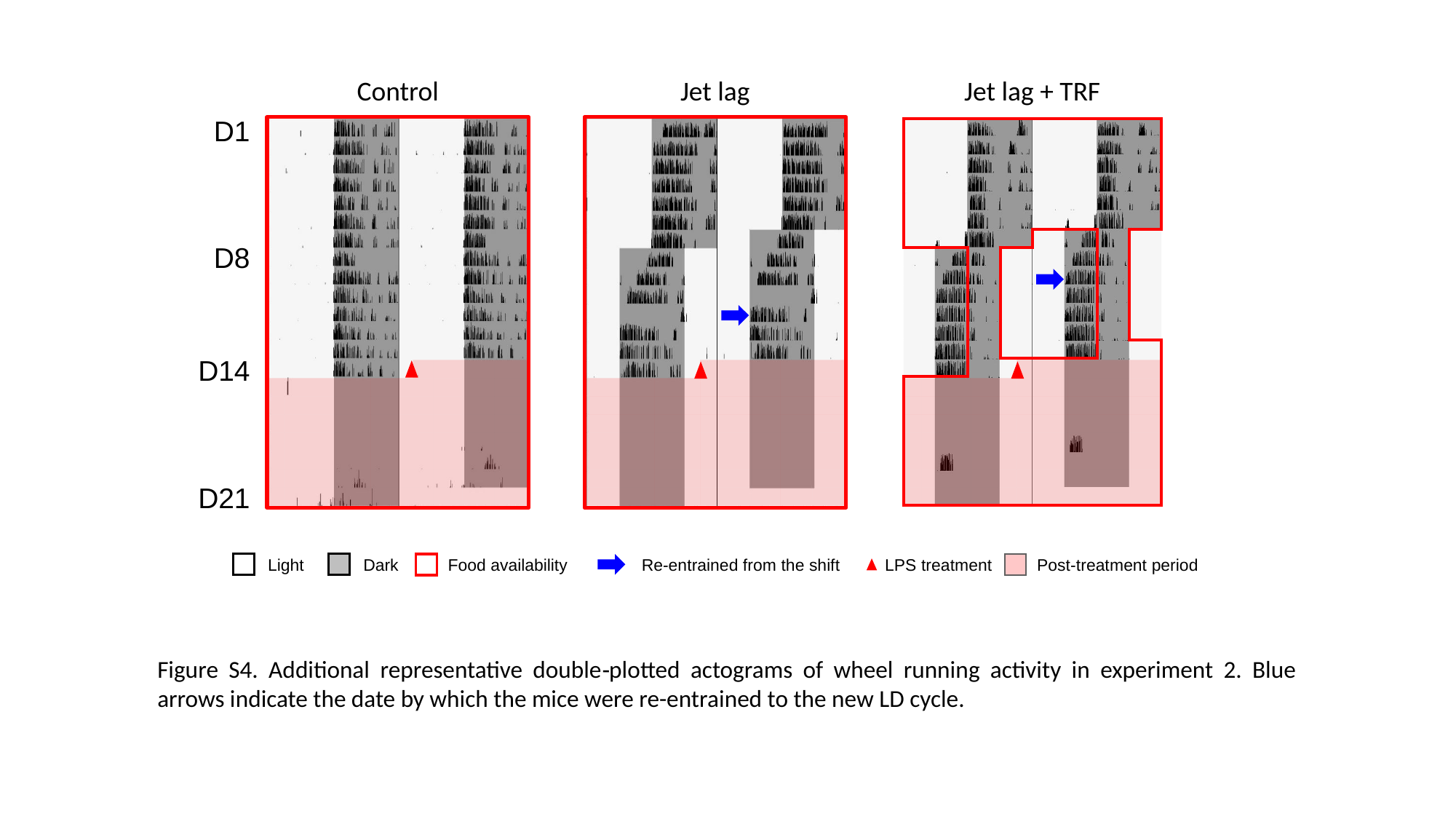

Control
Jet lag
Jet lag + TRF
D1
| | | | | | | | |
| --- | --- | --- | --- | --- | --- | --- | --- |
| | | | | | | | |
| | | | | | | | |
| | | | | | | | |
| | | | | | | | |
| | | | | | | | |
| | | | | | | | |
| | | | | | | | |
| | | | | | | | |
| | | | | | | | |
| | | | | | | | |
| | | | | | | | |
| | | | | | | | |
| | | | | | | | |
| | | | | | | | |
| | | | | | | | |
| | | | | | | | |
| | | | | | | | |
| | | | | | | | |
| | | | | | | | |
| | | | | | | | |
D8
| | | | | | | | |
| --- | --- | --- | --- | --- | --- | --- | --- |
| | | | | | | | |
| | | | | | | | |
| | | | | | | | |
| | | | | | | | |
| | | | | | | | |
| | | | | | | | |
| | | | | | | | |
| | | | | | | | |
| | | | | | | | |
| | | | | | | | |
| | | | | | | | |
| --- | --- | --- | --- | --- | --- | --- | --- |
| | | | | | | | |
| | | | | | | | |
| | | | | | | | |
| | | | | | | | |
| | | | | | | | |
| | | | | | | | |
| | | | | | | | |
| | | | | | | | |
| | | | | | | | |
| | | | | | | | |
| | | | | | | | |
| --- | --- | --- | --- | --- | --- | --- | --- |
| | | | | | | | |
| | | | | | | | |
| | | | | | | | |
| | | | | | | | |
| | | | | | | | |
| | | | | | | | |
| | | | | | | | |
| | | | | | | | |
| | | | | | | | |
| | | | | | | | |
D14
D21
Light
Dark
Food availability
Re-entrained from the shift
LPS treatment
Post-treatment period
Figure S4. Additional representative double‐plotted actograms of wheel running activity in experiment 2. Blue arrows indicate the date by which the mice were re-entrained to the new LD cycle.
